# Metabolic serum biomarkers for the prediction of cancer: a follow-up of the studies conducted in the Swedish AMORIS study

**DOI:** 10.3332/ecancer.2015.555

**Published:** 2015-07-23

**Authors:** Cecilia Bosco, Wahyu Wulaningsih, Jennifer Melvin, Aida Santaolalla, Mario De Piano, Rhonda Arthur, Mieke Van Hemelrijck

**Affiliations:** 1 King’s College London, Division of Cancer Studies, Cancer Epidemiology Group, Research Oncology, 3rd floor, Bermondsey wing, Guy’s Hospital, London SE1 9RT, UK; 2 Both authors contributed equally

**Keywords:** cancer, serum lipids, serum glucose, C-reactive protein, leukocytes, IgE, calcium, iron, gamma-glutamyl transferase

## Abstract

The Swedish Apolipoprotein MOrtality RISk study (AMORIS) contains information on more than 500 biomarkers collected from 397,443 men and 414,630 women from the greater Stockholm area during the period 1985–1996. Using a ten-digit personal identification code, this database has been linked to Swedish national registries, which provide data on socioeconomic status, vital status, cancer diagnosis, comorbidity, and emigration. Within AMORIS, 18 studies assessing risk of overall and site-specific cancers have been published, utilising a range of serum markers representing glucose and lipid metabolism, immune system, iron metabolism, liver metabolism, and bone metabolism. This review briefly summarises these findings in relation to more recently published studies and provides an overview of where we are today and the challenges of observational studies when studying cancer risk prediction.

Overall, more recent observational studies supported previous findings obtained in AMORIS, although no new results have been reported for serum fructosamine and inorganic phosphate with respect to cancer risk. A drawback of using serum markers in predicting cancer risk is the potential fluctuations following other pathological conditions, resulting in non-specificity and imprecision of associations observed. Utilisation of multiple combination markers may provide more specificity, as well as give us repeated instead of single measurements. Associations with other diseases may also necessitate further analytical strategies addressing effects of serum markers on competing events in addition to cancer. Finally, delineating the role of serum metabolic markers may generate valuable information to complement emerging clinical studies on preventive effects of drugs and supplements targeting metabolic disorders against cancer.

## Introduction

The Swedish AMORIS database is by far one of the largest prospective cohort studies with detailed information on serum biomarkers. Between 1985 and 1996, the Central Automation Laboratory collected and analysed blood samples of 397,443 men and 414,630 women, mainly from the greater Stockholm area [1–4]. All individuals were either healthy individuals referred for clinical laboratory testing as part of a general health checkup or outpatients. This database with information on >500 biomarkers has been linked to several Swedish national registries such as the National Cancer Register, the Patient Register, the Cause of Death Register, the consecutive Swedish Censuses during 1970–1990, and the National Register of Emigration. By using the Swedish ten-digit personal identity number one can get information on socioeconomic status, vital status, cancer diagnosis, comorbidity, and emigration.

With respect to cancer outcomes, 18 studies to date investigated the association with serum biomarkers of lipid and glucose metabolism, the immune system, liver metabolism, iron metabolism, and bone metabolism in AMORIS [5–22]. Following a brief overview of the results found for all biomarkers studied in AMORIS, the current review aims to summarise subsequently published epidemiological evidence on these serum biomarkers in relation to risk of cancer development.

## Literature review

For each following subsection we used related medical subject headings (MeSH) terms for the biomarkers studied in AMORIS as well as ‘neoplasm’. Both PubMed and Embase were searched only using the date of AMORIS publications as a limitation to ensure that we found all epidemiological evidence published subsequently to our findings in this Swedish prospective cohort. Studies relevant to previous work in AMORIS were selected and included in this review.

## Lipid metabolism

### Selected biomarkers

A wide variety of serum biomarkers allow the investigation into the association between lipid metabolism and cancer. **Triglycerides** constitute the majority of the lipids in the body, whereas **cholesterol** is a precursor for plasma membranes, bile salts, steroid hormones, and other specialised molecules. Cholesterol requires lipoproteins to be transported in the blood stream. **Low density lipoproteins** (LDL) are the main cholesterol carriers and they deliver cholesterol to cells throughout the body [23]. In contrast, **high-density** lipoproteins (HDL) remove excess cholesterol from blood and tissue. **Apolipoproteins A-I and B** (ApoA-I and ApoB) are structural proteins of these lipoprotein particles assisting in their transport [24].

Dyslipidaemia, or abnormal lipid metabolism, is thought to be involved in cancer development through a pathway linked to fatty acid synthesis [25–29]. High serum levels of lipid components such as triglycerides, total cholesterol, LDL, and ApoB have also been implicated in development of certain types of cancers such as breast and prostate by stimulating the Akt and AMPK pathways, which are associated with DNA damage and cell proliferation [30–32]. Additionally, hypercholesterolaemia has been shown to up-regulate the activity of transcriptional factors such as Sterol Regulatory Element-Binding Proteins (SREBP) and low-density lipoprotein receptor (LDLr), which promote carcinogenesis [33, 34]. All these evidence suggests a potential role of serum lipids in the prediction of cancer.

### Findings in AMORIS

We have studied the interplay between glucose, triglycerides, total cholesterol and the associated risk of prostate, kidney, and gastrointestinal cancers [10, 11, 14, 15]. Our findings supported the hypothesis that components from the lipid metabolism influence risk of developing cancer, although a greater risk of prostate cancer with increasing triglycerides was only seen in men with higher glucose levels [11].

Low levels of HDL and ApoA-I were also found to be associated with increased prostate cancer risk [14]. Additionally, we studied the link between serum lipids and risk of breast, endometrial, and ovarian cancer [7, 8], and found a positive association between serum triglycerides and risk of endometrial cancer, whereas only a weak inverse relation was observed for breast cancer.

### New epidemiological findings in the literature

Since the last AMORIS publication, several epidemiological studies have also focused on serum lipid markers and risk of prostate cancer (Table 1). A statistically significant positive association was observed with total cholesterol [35–38], whereas an inverse association was found for triglycerides [39]. When focusing specifically on aggressive prostate cancer, the Cancer Prevention Study II Nutrition Cohort [40] reported that neither total cholesterol, LDL- or HDL-cholesterol were associated with it. Also for gastrointestinal cancers, many more studies have been published. Total cholesterol and triglycerides have been positively associated with risk of colorectal cancer [41, 42], whereas HDL has been found to either have no effect or reduce this risk [43]. Most studies failed to demonstrate any effect of circulating lipids on risk of rectal cancer alone [43–45].

In addition, an increased risk for breast, bladder, and pancreatic cancer has been observed among those with high circulating levels of total cholesterol, triglycerides, LDL, and low circulating levels of HDL [35, 46–49] compared to those with normal levels. In contrast, no statistically significant association was found between lipid components and risk of ovarian cancer in the Metabolic syndrome and Cancer project (Me-Can) [50]. Similarly, null-findings were observed in a prospective cohort study based on a Korean population focusing on cervical, kidney, gall bladder, pancreatic, lung, and oesophageal cancers. However, in the same study when authors analysed serum lipid levels and the associated risk of stomach and liver cancer, they found an inverse association [35]. With respect to the inverse association between ApoA-I and cancer, as observed in AMORIS, four studies corroborated these findings [14, 43, 48, 49, 51].

### Where are we today?

Dyslipidaemia is closely linked to obesity, another emerging risk factor for several cancers [52]. This implies that despite the suggested mechanisms, abnormal lipid metabolism may be a proxy of other lifestyle-related factors underlying carcinogenesis. Nevertheless, there is evidence suggesting that statins, a class of lipid-lowering drug, may suppress cell proliferation and increase apoptosis by inhibiting the action of the enzyme hydroxymethylglutaryl coenzyme A (HMG-CoA) reductase [53–55], further indicating the involvement of lipids in carcinogenesis. The inverse association between ApoA-I and cancer as found in our study was potentially related to not only inflammation [56], but other lifestyle factors such as body mass index (BMI), cigarette smoking, alcohol intake, diabetes, or hypertension influencing the circulating levels of ApoA-I. This lipid biomarker has been shown to be predictive of cardiovascular risk [4, 57] and it is thus possible that the oetiological pathway between lipid profiles and atherosclerosis is different from the pathway between lipid profiles and cancer. The strong association between the lipid metabolism and cardiovascular disease also indicates a potential competing risk situation [58], where individuals at risk of cancer may die of cardiovascular disease before being diagnosed with cancer. This urges further studies to address the issue especially when assessing serum lipids in relation to cancer.

## Glucose metabolism

### Selected biomarkers

Disruptions in the glucose metabolism, which encompass an array of metabolic abnormalities such as diabetes, have been linked to chronic diseases including cancer [59]. Serum glucose is the most commonly measured marker of the **glucose** metabolism, representing current levels of glucose in the circulation. **Fructosamine** is another commonly used marker and reflects the average level of serum glucose in the previous 10–14 days [60]. Insulin, with elevated levels marking the initial stage of impaired glucose metabolism, has been suggested to be involved in carcinogenesis through its growth-promoting effects on cells [61]. Similar mutagenic effects have been suggested for a closely linked marker, insulin-like growth factor I (IGF-I) [62]. Additionally, serum glucose may directly affect cancer through generation of Advanced Glycation End-products (AGE), which leads to chronic inflammation [63]. Fructosamine, which represents all glycated serum proteins, may therefore also be involved in this mechanism. The role of impaired glucose metabolism in cancer development and survival has been suggested [64], for instance, Hammarsten *et al* showed in a prospective study of 320 prostate cancer patients that men who died of clinical prostate cancer during follow-up had a higher prevalence of type 2 diabetes (P < 0.035) and higher levels of fasting plasma insulin (P = 0.004) [65]. These results indicated that insulin levels could be used as markers of prostate cancer prognosis and tumour aggressiveness, regardless of the patient’s prostate cancer stage, cancer grade, and PSA level. Data from another prospective cohort in Sweden also suggested that insulin resistance related factors might be important for tumour progression [66]. With regards to breast cancer, two genetic variations (MNTR1a and 1b genes) have been shown to be associated both with cancer susceptibility and perturbed expression of insulin and glucose [67].

### Findings in AMORIS

Apart from the interplay between glucose, triglycerides, total cholesterol, we also investigated possible associations between glucose levels and risk of breast, endometrial, and ovarian cancer in a cohort of more than 230,000 women [7, 8, 21]. Our results indicated that glucose levels below diagnostic threshold for diabetes increased the risk of endometrial and postmenopausal breast cancer. Most recently, we investigated repeated measurements of glucose, and fructosamine in relation to cancer risk and found highest cancer risks for those in the highest tertile of glucose and lowest tertile of fructosamine [16].

### New epidemiological findings in the literature

The more recent literature provides further epidemiological evidence on how the glucose metabolism play a role in the risk of a number of cancers such as colon, liver, and endometrial cancers [68–72] (Table 2). Interestingly, one study on thyroid cancer found a positive association for serum glucose in men and an inverse association in women [73]. This might imply a different role of the glucose metabolism in endocrine-related cancers. However, studies on the role of serum glucose concentrations and breast cancer risk were inconclusive [74]. No new findings have been reported for the link between fructosamine and risk of cancer.

### Where are we today?

Common key players in impaired glucose metabolism and cancer may indicate that both share an underlying mechanism rather than any causal role of serum glucose in carcinogenesis [75]. However, a protective effect against cancer has been suggested for metformin, one of the main medications to lower blood glucose [76–78], which supports the role of the glucose metabolism. In addition to glucose-lowering effects, metformin also possesses a direct anti-tumour effect by inhibiting protein synthesis and cell proliferation [79]. Another issue to be addressed when assessing the glucose metabolism in relation to cancer is turnover times for the serum markers. Fructosamine and HbA1c, which remain in the circulation for a longer duration than serum glucose, may provide more accurate representation of individual glycaemic status. The variability of serum glucose may also be accounted for by using multiple measurements as performed in one of our studies [16], either as a cumulative average or time-varying covariates [80]. Future studies should also consider the role of glucose metabolism markers in other chronic diseases, which may distort its association with cancer.

## Immune system

### Selected biomarkers

The role of the immune system in carcinogenesis was first shown by an observation of cancer occurring in chronic inflammation [81]. It is thought that inflammation is capable of triggering both tumour initiation and promotion through the formation of reactive oxygen species (ROS) and reactive nitrogen intermediates (RNI) [82]. **C-reactive protein** (CRP) is one of the most investigated markers of inflammation in the context of cancer detection and prognosis. Higher levels of post-diagnosis CRP have been linked with worse survival rates in various malignancies [83–85]. In addition to CRP, **albumin, haptoglobin,** and **leukocytes** are other commonly used markers of inflammation. Albumin is an acute-phase protein involved in blood volume regulation and transportation of molecules of low water solubility such as lipid soluble hormones and calcium. Together with leukocytes, albumin has been studied as a marker of systemic inflammation in the context of cancer survival and so far results have shown that low levels of albumin and high levels of leukocytes are associated with worse cancer prognosis [86]. **Haptoglobin** is a positive acute-phase protein and its plasma levels increase during inflammatory processes such as infection, extreme stress, burns, major crush injury, or allergy. The full scope of the biological function of haptoglobin is not yet defined, however experimental studies have hypothesised that haptoglobin polymorphisms may contribute to increased oxidative stress and low-grade chronic inflammation [87, 88]. There is also observational evidence indicating that allergy (measured by **Immunoglobulin E** (IgE)), which is highly linked to inflammation, is associated with higher risk of solid tumours such as breast, prostate, and colorectum [9].

### Findings in AMORIS

We have studied different immunological markers in relation to cancer risk [9, 13, 22]. One study was of particular interest, because it replicated the findings for one measurement of CRP and leukocytes with three repeated measurements [13]. When looking into specific major cancers including prostate, breast, lung, gastrointestinal, bladder, cervix. and skin cancer, a positive association was only seen for lung cancer. The lack of association between inflammatory markers and specific cancer risk was further shown when we investigated serum CRP, leukocytes, albumin, and haptoglobin in relation to prostate cancer [22]. We also assessed the association between total serum levels of IgE and cancer risk in 24,820 persons and found a weak inverse association between quartiles of IgE and cancer risk [9].

### New epidemiological findings in the literature

A consistent association between serum CRP and cancer risk is corroborated by more recent findings (Table 3), as shown by a metaanalysis of 11 studies in Western populations showing an increased cancer risk for higher levels of CRP [89]. Similar findings were reported in Asian populations [90]. Results for specific cancers remain conflicting except for lung cancer, where a positive association with CRP and leukocytes has been reported. This is consistent with our findings seen in the AMORIS database [89, 91, 92]. Some evidence, although weaker, has been reported for colorectal, breast, ovarian. and liver cancer [93–97], whereas no association has been found for prostate and pancreatic cancers [98–100]. Regarding serum IgE, most observational studies confirmed an inverse association with risk of developing brain cancer, particularly glioma [101–104]. To date, little evidence exists for association with any other cancers.

### Where are we today?

Although biological studies consistently link inflammation to carcinogenesis [105], the role of common serum inflammatory markers in predicting cancer risk still remains unclear. This may be partly because of the wide spectrum of inflammation, which is also an essential part of many pathologic conditions such as cancer and cardiovascular disease. The non-specificity of such cancer markers may explain the lack of associations found in observational studies, urging future studies to deploy novel methods to increase sensitivity of cancer prediction using these markers. Another possible explanation is the genetic variation of these markers, instead of their quantitative protein expression, that influences cancer development. This is supported by two recent studies suggesting different risk of colorectal cancer conferred by CRP polymorphisms [106, 107]. Additionally, these markers are usually analysed separately and a combined analysis may provide a better approximation with respect to early cancer detection, as it has shown in the case when combining scores of CRP with IL-8 [108] or haptoglobin with serum amyloid A (SAA) [109] in predicting lung cancer risk, and the ratio of reactive oxygen metabolites and CRP for colorectal cancer [110].

## Liver metabolism

### Selected biomarkers

**Gamma-glutamyl transferase** (GGT), is a central enzyme in the glutathione (GSH) metabolism, a ubiquitous antioxidant thiol, and plays an important role in maintaining tissue oxidant/antioxidant balance, cellular defence, proliferation, and protection against further oxidative stress [111]. The latter may explain its potential role in carcinogenesis, in addition to its links with type 2 diabetes, cardiovascular, and chronic kidney disease [112–115]. Elevated levels of GGT have been associated with poorer endometrial cancer prognosis, increased risk of progression of high-grade cervical dysplasia to invasive carcinoma [116], increased risk of breast cancer amongst premenopausal women [117], increased risk of cancer in men [118], increased risk of liver cancer [119] and it has been reported to play an independent role in the prediction of overall survival (OS) in metastatic colorectal carcinoma [120].

### Findings in AMORIS

We have investigated GGT serum levels in relation to cancer risk in 545,460 persons and found evidence of associations between elevated GGT and risk of developing different cancers. The strength of this association varied by levels of glucose which may suggest that hyperglycaemia can result in oxidative stress which in turn initiate damaging pathways of carcinogenesis [19].

### New epidemiological findings in the literature

Since the last AMORIS publication, several studies have analysed the association between GGT and cancer risk and prognosis [121–128] (Table 4). All studies are in agreement with our findings in AMORIS and show that high levels of GGT are an indicator of elevated cancer risk and poor disease prognosis. Three studies showed that high pre-therapeutic levels of GGT are associated with advanced tumour stage and serve as an independent prognostic marker of poor prognosis in gynaecological cancers [122, 125, 126]. A case-cohort study in Taiwanese men showed that high levels of GGT were associated with risk of all-cause death, all cancer, and hepatocellular carcinoma (HCC) mortality [124]. Furthermore, another study analysing GGT and HCC prognosis showed that high levels of pre-treatment GGT were associated with reduced OS rates, when compared to those with normal pre-treatment GGT levels [121]. In addition, elevation of serum GGT levels was found to be an indicator of aggressive intrahepatic cholangiocarcinoma behaviours and a predictor of poor clinical outcomes [127]. Interestingly, one study in Japanese adults found that GGT was only a predictor of cancer risk for alcohol-related cancers in current drinkers [123]. GGT has also been reported to play an independent role in the prediction of OS in metastatic colorectal carcinoma [120].

Finally, a meta-analysis by Long *et al* concluded that GGT predicts cardiovascular and cancer mortality [129], whereas Kunustor *et al* in their meta-analyses showed that baseline levels of GGT are positive independent predictors of overall cancer risk as well as for all-cause mortality [130, 131].

### Where are we today?

Overall epidemiological evidence shows that high levels of GGT are associated with cancer risk and many experimental studies have intended to explain this link suggesting different biological mechanisms [132–136]. These pathways have been demonstrated for cancer specific sites which may be explained by the high variability present in cancer cells together with the effect of other factors, such as environment, drugs, and diet that could modify cancer cells phenotype including GGT expression [137].

## Iron metabolism

### Selected biomarkers

The iron metabolism is another pathway potentially linked with carcinogenesis. Iron plays a fundamental role in important biological processes in eukaryotic cells such as oxygen transport, cellular respiration, and redox reactions; consequently iron homeostasis is precisely regulated. Most **circulating iron** is bound to transferrin; the rest of iron is either serum-free iron or iron stored in cells bound to ferritin. **Total iron-binding capacity (TIBC)** measures the ability of plasma proteins to bind iron and reflects the fraction of transferring- free places to bound iron, meaning that low values of TIBC evidence transferrin saturation (TSAT) and consequently high iron stores in cells.

Different mechanisms of iron involvement in carcinogenesis have been suggested, including oxidative DNA damage by iron-catalysed free radical production, alterations in gene expression consistent with increased iron requirements in proliferating cells, as well as decreased immune surveillance against cancer [138]. Excess iron has been shown to promote protein and genomic alterations mirrored in human cancers [139] and this may occur via iron-induced persistent oxidative stress [139]. Moreover, iron sequestration machinery is activated by inflammatory processes associated with chronic diseases such as breast cancer for which cancer-associated anaemia is being broadly studied [140].

### Findings in AMORIS

Using a cohort of 220,642 participants with baseline measurements of serum iron, TIBC, and CRP, we found a positive association between TIBC (i.e. low TSAT) and the risk of overall and in particular colon cancer [5]. Serum iron, on the other hand, did not correlate with overall cancer risk, although a positive association with postmenopausal breast cancer was shown. These observations thus support a role of iron metabolism in relation to specific cancer risk.

### New epidemiological findings in the literature

Only one recently published study focused on serum iron as a marker of the iron metabolism in the context of cancer risk. This cohort study of 309,443 men and women in Taiwan reported an increased risk of cancer in individuals with high serum iron [141]. Specific cancer analysis showed an increased risk of breast cancer for serum iron ≥140 μg/dL—hazard ratio (HR): 1.31 95%, confidence interval (CI): 1.01–1.70—compared to lower levels, which is similar to our findings for postmenopausal breast cancer. Other recent studies measured iron based on dietary intake subclassified as dietary heme iron, supplemental iron, and dietary intake of meat [142–145]. Dietary iron was assessed mainly using food frequency questionnaires and heme iron intake was usually determined indirectly by calculating a type-specific percentage of the total iron content in meat [144, 145]. Furthermore, a broad meta-analysis examining different cancer types in association with serum iron markers and dietary iron markers, found a negative association between cancer risk and levels of iron storage biomarkers, mostly with serum ferritin. Moreover, authors reported that a higher intake of heme iron showed a tendency towards a positive association with cancer risk [146]. Similar conclusions for dietary markers were obtained in a colorectal cancer meta-analysis, suggesting a significant positive association of heme iron intake and risk of colorectal cancer [147].

### Where are we today?

Iron homeostasis is closely linked to anaemia, which impairs many physiological processes [148]. Considering the association between anaemia and mortality [149], it is possible that the positive association between serum iron and risk of cancer emerges as a consequence of other fatal diseases in persons with low levels of iron, thus removing them from the population at risk of developing cancer. Future research should address risks associated with different types of anaemia in addition to serum components of iron metabolism when assessing their link to cancer susceptibility.

## Bone metabolism

### Selected biomarkers

Components of bone metabolism have been indicated to be involved in carcinogenesis. Since calcium homeostasis is mainly influenced by vitamin D and parathyroid hormone instead of dietary calcium [150], the use of serum **calcium** could be useful in investigating the aetiology of cancer. Ionised serum calcium level is a direct measure of the amount of metabolically active serum calcium but is not routinely measured [151]. **Correction of total calcium levels based on serum albumin** is therefore used to obtain an estimate of the free ionised calcium level, since almost half of serum calcium is in protein-bound form and alteration of serum albumin may affect levels of free ionised calcium [150, 151]. **Inorganic phosphate** (Pi) is another dietary constituent well-known for its role in skeletal mineralisation, and normal levels of Pi are essential to maintain normal cellular function [152]. As a result, it has been suggested that Pi may act as an active regulator of growth rather than a merely compulsory element in cellular homeostasis. A particular link between calcium and gastrointestinal cancer has been suggested, since dietary calcium may activate calcium receptor and bind bile acids in gastrointestinal tract, in addition to the role of serum calcium in cellular metabolism [153, 154]. Recent studies also indicated that inorganic phosphate might be implicated in carcinogenesis, as high-inorganic phosphate diet has been linked to an increased development of lung and skin cancers [155, 156]. Abnormal levels of inorganic phosphate are thought to affect carcinogenesis by amplification of Akt signalling and 5’ cap eukaryotic dependent translation [157, 158].

### Findings in AMORIS

We investigated serum calcium in relation to risk of prostate and gastrointestinal cancer, and serum inorganic phosphate in relation to risk of overall and site-specific cancers [5, 17, 18, 20]. We found a weak negative association between calcium and prostate cancer, which was likely explained by a strong association between calcium and all-cause mortality. For gastrointestinal cancer, higher risks of oesophageal and colorectal cancer were linked to higher levels of albumin-corrected calcium in women, indicating the importance of calcium correction based on albumin levels. In men, a similar but weaker association was found. The study focusing on inorganic phosphate showed a positive association with risk of overall cancer in men, but an inverse association in women.

### New epidemiological findings in the literature

In support of the above findings, another Swedish-based study showed a positive trend between levels of albumin-adjusted calcium and risk of prostate cancer in men [159] (Table 5). Similar findings with total and ionised serum calcium were reported when prostate cancer death was used as a surrogate outcome [160]. Nonetheless, an inverse association was observed in an Asian study [161]. No new studies have been published investigating the association between serum inorganic phosphate and risk of cancer.

### Where are we today?

In clinical studies, the potential chemopreventive effects of calcium in cancer, particularly colorectal cancer, remain conflicting [162]. A recent dose-response meta-analysis showed an inverse association between dietary calcium, calcium supplementation, and risk of colorectal cancer [163]. However, the role of serum levels of calcium as well as its counterpart, serum inorganic phosphate, in relation to cancer prediction remains elusive. As bone metabolism is tightly regulated, abnormalities in calcium and phosphate levels may reflect a defect in bone regulation instead of dietary intake. Further clinical and observational studies exploring the potential roles of calcium and phosphate in cancer should take into account their regulators such as vitamin D, parathyroid hormone, and fibroblast growth factor 23 (FGF-23) [164, 165] in order to fully comprehend how they are involved in carcinogenesis.

## Conclusion

Overall, more recent observational studies supported previous findings obtained in AMORIS, although no new results have been reported for serum fructosamine and inorganic phosphate with respect to cancer risk. A drawback of using serum markers in predicting risk of cancer is its potential fluctuations following other pathological conditions, resulting in non-specificity and imprecision of associations observed. Utilisation of multiple combination markers may provide benefit from enhanced specificity in relation to cancer, as well as repeated or serial measurements instead of a single measurement. Associations with other diseases may also necessitate further analytical strategies addressing effects of serum metabolic markers on competing events in addition to cancer. Finally, delineating the role of serum metabolic markers may generate valuable information to complement emerging clinical studies on preventive effects of drugs and supplements targeting metabolic disorders against cancer.

## Figures and Tables

**Table 1.** Epidemiological studies on lipid metabolism and cancer.

Publication	Study population	Study design	No. Of subjects, follow-up	Exposure	Outcome	Main results	Adjustments
Haggstrom, H. 2012 [39]	Me-Can cohort	Prospective cohort	289,866 men included.	Smoking status, BMI, blood pressure, glucose, cholesterol, and TG.	PCa risk	High levels of triglycerides were associated with a decreased risk of pca top quintile RR 1.24 (1.06–1.45) bottom quintile 0.88 (0.74–1.04).	Smoking, BMI.
Jacobs, E.J.2012 [40]	Cancer prevention study II nutrition cohort	Cohort.	236 cases and 236 matched controls.	TC, LDL cholesterol, HDL cholesterol, non-HDL cholesterol. (non-fasting).	PCa risk	Neither total, LDL, nor HDL cholesterol concentrations were associated with risk of pca. OR 0.93 (95% CI 0.76–1.14) for total cholesterol and 0.97 (95% CI 0.82–1.16)	Age, race, blood draw date, physical activity, use of cholesterol-lowering drugs, and history of heart attack.
His, M 2014. [49]	Supplementation en vitamines et mineraux antioxydants study	Cohort	7557 subjects	TC, LDL cholesterol, HDL cholesterol, TG, ApoA1, apob	Breast cancer and PCa risk	TC was inversely associated with overall (HR = 0.91 95% CI 0.82–1.00) and breast (HR = 0.83 95% CI 0.69–0.99) cancer risk. HDL-c was also inversely associated with overall (HR = 0.61 95% CI 0.46–0.82) and breast (HR = 0.48 95% CI 0.28–0.83) cancer risk. Consistently apoa1 was inversely associated with overall (HR = 0.56 95% CI 0.39–0.82) and breast (HR = 0.36 95% CI 0.18–0.73) cancer risk.	Age, intervention group, number of dietary records, alcohol intake per day, physical activity. Smoking status, educational level, height, BMI, family history of bca, menopausal status at baseline, TG-lowering drugs antihypertensive drugs, energy intake per day and glycaemia. Ratio models adjusted for TG and TC.
Wu, Q. 2012 [48]	Hospital PUMCH patient information database	Case-control	210 pancreatic adenocarcinoma, 630 healthy controls	TC, LDL cholesterol, HDL cholesterol, TG, ApoA1, apob, fasting blood glucose.	Pancreatic adenocarcinoma risk	TC (OR–1.793 95% 1.067–3.013) and ApoA (OR = 36.065 95% 15.547–83.663) were significantly related to pancreatic adenocarcinoma.	Age and sex.
Agnoli, C. 2014 [41]	Colorectal cancer cases	Cohort	1134 participants 850 in randomly selected cohort and 286 colorectar cancer cases	TC, LDL cholesterol, HDL cholesterol, TG. (Fasting)	Colorectal cancer risk	Highest tertiles of total (HR = 1.66 95% 1.12–2.45) and LDL cholesterol (HR1.87 95% CI 1.27–2.76) were associated with increased colorectal cancer risk.	Age, gender, BMI, smoking, total physical activity, alcohol consumption, dietary red meat, dietary fiber, and dietary calcium.
Jiang, R. 2014 [51]	Cancer registry	Cohort	807 patients.	TC, LDL cholesterol, HDL cholesterol, TG, ApoA1, ApoB,	Nasopharyngeal carcinoma survival	ApoA-I levels (HR = 0.64 95% CI 0.52–0.80) were associated with a favourable OS.	Adjustment for clinical characteristics and other serum lipids and lipoproteins
Kim, H.S.2013 [42]		Cohort	14932	BMI, H.pylori, TC, LDL-c, HDL-c, TG	Prevalence and risk factors of colorectal cancer	Predictor of colorectal cancer was hypertriglyceridemia (OR = 1.267 95% CI 1.065–1.508)	–
Shafique, K. 2012 [38]	Midspan studies	Prospective cohort study	12,926 men (650 cases)	Baseline cholesterol	Incidence of pca and prognosis	Baseline plasma cholesterol was associated with hazard of high grade PCa incidence (n = 119).	Association remained significant after adjustment for body mass index, smoking and socioeconomic status
Kitahara *et al* 2011 [35]	Korean adults enrolled in the National Health Insurance Corporation	Cohort	53,944 men and 24,475 women	TC (fasting)	Cervix, breast, colon, lung, pancreas, bladder, kidney, oesophagus, gall bladder, liver, rectal, prostate cancer risk	TC (≥ 240 mg/dL) was associated with PCa (HR 1.24; 95% CI, 1.07 –1.44; P = 001) and colon cancer (HR, 1.12; 95% CI, 1.00–1.25; P = 05) in men. Breast cancer (HR, 1.17; 95% CI, 1.03 –1.33; P trend = 03). Total cholesterol was inversely associated with all-cancer incidence in both men (HR, 0.84; 95% CI, 0.81–0.86; P < 001) and women (HR, 0.91; 95% CI, 0.87–0.95; P < .001).	Adjustments for cigarette smoking, alcohol consumption, BMI, physical activity, hypertension and fasting serum glucose .
Mondul *et al*, 2011 [37]	ATBC Study	Cohort	2041	TC, HDL (fasting)	PCa risk	Men with higher serum TC were at increased risk of overall (≥ 240 versus <200 mg/dl: HR = 1.22, 95% CI 1.03–1.44, ptrend = 0.01) and advanced (≥240 versus<200 mg/dL: HR = 1.85, 95% CI 1.13–3.03, p-trend = 0.05) prostate cancer	Adjusted for serum α-tocopherol, family history of prostate cancer, education level, and urban residence, other cholesterol type, smoking habits, BMI, marital status; total energy, total fat, fruit, vegetable, red meat, alcohol, dietary retinol, vitamin D, calcium intake. Subgroup analyses were conducted stratifying by follow-up time (<ten years, >ten years).
Kok *et al*, 2011 [36]	Nijmegen Biomedical Study	Cohort	2842	TG, TC, HDL, LDL	PCa risk	Higher total and higher LDL cholesterol were significantly associated with an increased risk of prostate cancer HR 1.39 (95% CI 1.03–1.88) and 1.42 (95% CI 1.00–2.02), respectively. Similar results were observed for aggressive prostate cancer, whereas for non-aggressive prostate cancer a significant association with HDL cholesterol was found HR 4.28, 95% CI 1.17–5.67.	Adjusted for age, body mass index and history of diabetes mellitus
Agnoli *et al*, 2010 [47]	Cancer registry	Cohort	163	TG, HDL	Breast cancer risk	Metabolic syndrome associated with breast cancer risk (rate ratio 1.58 [95% confidence interval 1.07–2.33]), Low serum HDL-cholesterol and high triglycerides were significantly associated with increased risk	Adjusted for matching variables and for: age, age at menarche, years from menopause, number of full-term pregnancies, age at first birth, oral contraceptives, hormone therapy, years of education, history of breast cancer in first degree relatives, breastfeeding, smoking, and alcohol consumption.
Bjorge *et al*, 2011 [50]	Me-Can study	Cohort	644	TG, TC (fasting and non-fasting)	Ovarian cancer	–	Year of birth, age at measurement, smoking and quintile levels of BMI
Van Duijnhoven *et al*, 2011 [43]	EPIC study	Nested case-control (EPIC)	1238	TG, TC, HDL, LDL, Apo A-1, Apo B (NS)	Colorectal cancer risk	HDL and apoA were inversely associated with the risk of colon cancer (RR for 1 SD increase of 16.6 mg/dl in HDL and 32.0 mg/dl in apoA of 0.78 (95% CI 0.68–0.89) and 0.82 (95% CI 0.72-0.94), respectively.	Height, weight, smoking habits, physical activity, education, consumption of fruit, vegetables, meat, fish and alcohol, intake of fibre, energy from fat and energy from non-fat
Hu *et al*, 2011 [43]	Cancer registry	Case-control	397	TG, HDL (fasting)	Colorectal cancer risk	TGs associated with cancer risk ·HR for ≥150mg/dl vs <150mg/dL:1.18; 95% CI: 0.9–1.51. HDL (-):· HR for < 40mg/dL versus ≥40mg/dL (men) or <50 mg/dL versus ≥ 50mg/dL (women): 0.94; 95% CI: 0.71–1.24.	Age, sex, smoking, drinking, past history of adenoma, other components of metabolic syndrome.
Aleksandrova *et al* 2011 [45]	EPIC study	Nested case-control(EPIC)	689	TG, HDL, (fasting and non-fasting)	Colon, rectal, cancer risk	Reduced HDL associated with colon cancer risk RR for ≤ 40 mg/dL versus > 40mg/dL in men and ≤ 50mg/dL versus > 50mg/dL in women: 1.36; 95% CI: 1.04–1.77.	Smoking status, education, alcohol consumption, physical activity, fiber intake, consumption of fruits and vegetables, red and processed meat, fish, and shellfish.
Stocks *et al*, 2011 [46]	Me-Can study	Cohort	2834 men, 1861 women	TG, TC (fasting and non-fasting	Colorectal cancer risk	TGs were found to be positively associated with cancer risk RR for fifth versus first quintile: 1.65; 95% CI: 1.27–2.13 (men), RR for fifth versus first quintile: 1.42; 95% CI: 1.09–1.85 (women).).	Smoking, five categories of birth year, age at measurement and quintiles of BMI

**Table 2.** Epidemiological studies on glucose metabolism and cancer.

Publication	Study population	Study design	No. of subjects, follow-up	Exposure	Outcome	Main results	Adjustments
Parekh, 2013 [68]	The Framingham Offspring Cohort, USA, men and women, age 20+ years	Cohort	3707 without cancer, duration 37 years	Fasting serum glucose	Obesity-related cancers	HR: 1.57 (95% CI: 1.17–2.11) for fasting glucose >110 mg/dLversus lower(measured 20+ yearsprior to censoring time)	Adjusted for age, sex, alcohol, smoking, and BMI. Obesity-related cancers were defined as cancers of the gastrointestinal tract, reticuloendothelial systems, female reproductive tracts, genitourinary organs, and the thyroid gland. Similar increased risk for colon cancer
Boyle, 2013 [74]	USA, Austria, Sweden, Korea, Italy	Meta-analysis	Six cohort, three case control, one case cohort studies	Serum glucose	Breast cancer	Summary RR: 1.11 (95 % CI: 1.00–1.23)	I^2^: 0 %
Friedenrich, 2012 [69]	Canada, women, mean age 59 (cases) and 59 (controls)	Case control	514 cases, 962 controls	Serum glucose	Endometrial cancer	OR: 1.26 (95% CI: 1.11–1.43) for every unit increase	Matched on age groups. Adjusted for age
Ulmer, 2012 [70]	Metabolic syndrome and cancer project (Me-Can), Austria, Norway, Sweden, women, mean age 44.1 years	Cohort	288274 without cancer, mean FU 11.3 years	Serum glucose	Cervical cancer	HR: 0.62 (95% CI: 0.20–1.96) for the highest versus lowest quintile	Stratified by centre, sex, and year of birth. Adjusted for age, smoking
Borena, 2011 [71]	Metabolic syndrome and cancer project (Me-Can), Austria, Norway, Sweden, men and women, mean age 43.9 (men) and 44.1 (women)	Cohort	406364 without cancer, mean FU 12.8 years (men) and 11.3 years (women)	Serum glucose	Liver cancer (primary)	RR: 2.38 (95% CI: 1.76–3.14) for every log unit increase	Stratified by cohort, birth year, and sex. Adjusted for age, smoking
Almquist, 2011 [73]	Metabolic syndrome and cancer project (Me-Can), Austria, Norway, Sweden, men and women, mean age 43.9 (men) and 44.1(women)	Cohort	578700 without cancer, mean FU not specified	Serum glucose	Thyroid cancer	RR: 9.24 (95% CI: 1.46–59.6) in men, 0.16 (0.01–0.69) in women, for the highest versus lowest quintile	Stratified by cohort, age. Adjusted for BMI, smoking, age
Johansen, 2010 [72]	Metabolic syndrome andcancer project (Me-Can), Austria, Norway, Sweden, men and women, meanage 43.9 (men) and 44.1 (women)	Cohort	577315 without cancer, mean FU 12.8 years (men) and 12.8 years (female)	Serum glucose	Pancreatic cancer	RR: 2.05 (95% CI: 0.84–4.94) for the highest versus lowest quintile	Stratified by cohort, birth year. Adjusted for BMI, smoking, age

**Table 3.** Epidemiological studies on Immune system and cancer.

Publication	Study population	Study design	No. of subjects, follow-up	Exposure	Outcome	Main results	Adjustments
Guo, 2013 [89]	USA, UK, Denmark, Sweden	Meta-analysis	194796 total participants, 11459 cancer	CRP	Overall cancer	Summary RR: 1.11 (95% CI: 1.03–1.18)	P_heterogeneity_ < 0.0001, I^2^ = 70%
Lee, 2011 [90]	South Korea, men and women, mean age 55 in cases and 47 in noncases	Cross-sectional	80781 without cancer, mean FU	CRP	Overall cancer	OR: 1.94 (95% CI: 1.51-2.51) for CRP >3 versus< 1 mg/L)	Adjusted for age, sex, BMI, diabetes,hypertension, dyslipidemia, smoking, alcohol consumption, exercise, aspirinuse, education level, and income
Xu, 2013 [92]	China, men and women, age 36–68 years	Case-control	96 cases, 124 controls	CRP	Lung cancer	OR: 2.11 (95 % CI, 1.66–2.91) for highest quartile versus lowest	Adjusted for smoking, gender, height, age, race, BMI, education, occupation, and living place
Dossus, 2014 [93]	The E3N prospective cohort, France, women, born between 1925–1950	Case control	549 cases, 1040 controls	CRP	Postmenopausal breast cancer	OR: 1.24 (96% CI: 0.92–1.66) for CRP 2.5–10 mg/L versus< 1.5 mg/L	Adjusted for matching variables: age at blood collection, menopausal status at blood collection, year of blood collection, centre of collection, and age at menopause
Toriola, 2013 [94]	Women’s Health Initiative Observational Study (WHI-OS), USA, women, age 50–79 years	Case control	988 cases, 988 controls	CRP	Colorectal cancer	OR: 1.30 (0.93–1.82) for highest quintile versus lowest	Matched on age, race, centre, date of blood-draw, baseline hysterectomy status. Adjusted for age, BMI, hormone replacement therapy, previous colonoscopy, pack-years of smoking use
Toriola, 2013 [100]	the Kuopio Ischemic Heart Disease Risk Factor Study (KIHD), Finland, men, age 42–60 years	Cohort	203 free from cancer, mean FU 24 years	CRP	Prostate cancer	1.08 (95% CI: 0.74–1.60) for highest tertile versus lowest	Adjusted for age, examination year, socioeconomic status, alcohol consumption, energy in take, cardiorespiratory fitness, BMI and smoking
Toriola, 2011 [97]	The Finnish Maternity Cohort (FMC), Finland, women, mean age 28.6 (cases) and 28.7 (controls)	Case control	91 cases, 115 controls	CRP	Ovarian cancer	OR: 1.62 (0.93–2.83) for highest tertile versus lowest	Adjusted for age
Trabert, 2014 [95]	The Prostate, Lung, Colorectal, and Ovarian Cancer Screening Trial, USA, women, age 55–74 years	Case control	149 cases, 149 controls	CRP	Ovarian cancer	OR: 2.04 (1.06–3.93) for highest tertile versus lowest	Matched on age, race, study centre, time and date of blood collection. Adjusted for BMI, smoking, parity, duration of oral contraceptive use, and duration of menopausal hormone therapy use
Aleksandrova, 2014 [96]	The European Prospective Investigation into Cancer and Nutrition (EPIC), Europe, men and women, 35–75 years	Case control	125 cases, 250 controls	CRP	Hepato cellular carcinoma	RR: 1.22 (1.02–1.46) per doubling of serum level	Matched on study center, sex, age, date of blood collection, fasting status, and time of blood collection. Women were additionally matched on menopausal status and exogenous hormone use. Adjusted for education, smoking, alcohol, diabetes, coffee, HBsAg/anti-HCV, BMI and waist to height ratio (WHtR)
Bao, 2013 [98]	The Health Professionals Follow-up Study (HPFS), the Nurses’ Health Study (NHS) the Physicians’ Health Study I (PHS I), the Women’s Health Initiative (WHI), the Women’s Health Study (WHS), USA,	Case control	491 cases, 1137 controls	CRP	Pancreatic cancer	OR: 0.99 (0.98–1.01) for every unit increase	Matched on year of birth, prospective cohort (which concurrently matched on sex), smoking status, fastingstatus, and month of blood draw. Adjusted for race, history of diabetes, BMI, physical activity, current vitamin use, levels of vitamin D and C-peptide
Grote, 2012 [99]	The European Prospective Investigation into Cancer and Nutrition(EPIC), Europe, men andwomen, 35–75 years	Case control	455 cases, 455 controls	CRP	Pancreatic cancer	OR: 1.01 (0.92–1.11) per doubling of serumlevel	Matched on recruitment centre, sex, age, date at entry, time between blood sampling and last consumption of foods and drinks, hormone use. Adjusted for smoking and BMI
Calboli, 2011 [101]	The Health Professionals Follow-up Study (HPFS), the Nurses’ Health Study (NHS), the Physicians’ Health Study (PHS), the Women’s Health Study (WHS), USA,	Case control	169 cases, 520 controls	Total IgE	Glioma	OR: 0.97 (0.88–1.07) for every unit increase	Matched on year of birth, cohort (which automatically matches the sex), month of blood collection, andethnic background.
Schlehofer, 2011 [102]	The European Prospective Investigation into Cancer and Nutrition (EPIC),Europe,	Case control	696 cases, 1188 controls	Allergen-specific IgE	Glioma	OR: 0.73 (0.51–1.06)for positive versus negative	Matched on study centre, sex, date of birth, age, date and time of blood collection , length of follow-up. Adjusted for education and smoking. Similar non statistically significant results for meningioma and schwannoma
Schwartzbaum, 2012 [103]	The Janus Serum Bankcohort, Norway, men and women, age 35–49 years	Case control	594 cases, 1177 cases	Allergen-specific IgE	Glioma	OR: 0.95 (0.75–1.22) for positive versus negative	Matched on two-year age interval,sex, and date of blood collection
Wiemels, 2011 [104]	USA, men and women, age 20–79 years	Case control	61 cases, 192 controls	Total IgE	Meningioma	OR: 0.85 (95% CI:0.75–0.98	Matched on five-year age interval, sex, and state of residence. Adjusted for sex, race, smoking, age, education

**Table 4.** Epidemiological studies on liver metabolisms and cancer.

Publication	Study population	Study design	No. of subjects, follow-up	Exposure	Outcome	Main results	Adjustments
Zhang *et al* 2011 [121]	Cancer registry	Cohort	277	GGT	Hepatocellular carcinoma prognosis	The one-year and three-year OS rates were 71.6 and 38.5% in patients with normal GGT and 48.8 and 16.9% in patients with high GGT (P = 0.002).	–
Yin *et al* 2013 [127]	Cancer registry	Cohort	411	GGT	Intrahepatic cholangiocar-cinoma prognosis	GGT was an independent predictor of a poor prognosis (hazard ratio =2.36, 95% confidence interval: 1.67–3.34, P = 0.001)	–
Tsuboya *et al* 2012 [123]	Ohsaki Cohort Study	Cohort	15 031	GGT	Overall cancer incidence	Highest quartile (GGT ≥31.0 IU/mL), the multivariate HR for any cancer was 1.28 (95% CI, 1.08–1.53; P for trend, <0.001), the HR for colorectal cancer was significantly greater than unity. This positive trend was observed only in current drinkers	Adjusted for age sex, drinking habit, self-reported history of liver disease, smoking habit body mass index, education, exercise.
Seebacher *et al* 2012 [122]	Multicenter database	Multicenter trial	874	GGT	Endometrial Cancer prognosis	Elevated serum GGT levels (P = 0.03 and P = 0.005), tumour stage (P < 0.001 and P < 0.001), grade (P < 0.001 and P = 0.02) and age (P < 0.001 and P < 0.001) were independently associated with progression-free survival in univariate and multivariable survival analyses	Patients were stratified in GGT risk groups
Hofbauer *et al* 2014 [128]	Cancer registry	Cohort	921	GGT	Renal cell carcinoma prognosis	Gamma-glutamyltransferase levels increased with advancing T (P < 0.001), N (P¼ 0.006) and M stages (Po0.001), higher grades (P < 0.001), and presence of tumour necrosis (Po0.001). An increase of GGT by 10Ul 1 was associated with an increase in the risk of death from RCC by 4% (HR 1.04, P < 0.001).	Adjusted for T stage, N stage, M stage, Fuhrman grade, necrosis histologic subtype.
Hernaez *et al* 2013 [124]	MJ Health Study	Case-Cohort	3961	GGT	Hepatocellular carcinoma mortality	High levels of GGT were associated with cancer mortality (HR1.8–2.8) and HCC mortality (HR 5.5–36.1).	Adjusted for age at baseline, body mass index, physical activity, smoking and alcohol use, education, systolic and diastolic blood pressure, total cholesterol, HDL, C-reactive protein HBsAg
He *et al* 2013 [120]	Cancer registry	Cohort	239	GGT	Colorectal Carcinoma prognosis	GGT (P < 0.001) statistically significant prognostic factor of overall survival validated as independent predictor. On univariate analysis, GGT (P < 0.001) statistically significant predictive factor of progression-free survival (PFS) in patients having first-line chemotherapy	–
Grimm *et al* 2013 [125]	Cancer registry	Multicenter study	634	GGT	Ovarian cancer prognosis	High GGT serum levels were associated with advanced FIGO stage (P < 0.001) and with worse overall survival in univariate (P < 0.001) and multivariable analysis (P = 0.02, HR 1.2 (1.1–1.5)	Adjusted for continuous GGT values and survival
Edlinger *et al* 2013 [126]	Vorarlberg Health Monitoring and Promotion Programme	Sub-Cohort	318	GGT	Endometrial cancer prognosis	GGT associated with cancer-related mortality (HR = 3.35, 95% CI 1.12–10.03)	Adjusted for age, tumour-staging (FIGO) and histology, together with the examination year, body mass index, hypertension, triglycerides, total cholesterol, glucose.

**Table 5.** Epidemiological studies on bone metabolism and cancer.

Publication	Study population	Study design	No. of subjects, follow-up	Exposure	Outcome	Main results	Adjustments
Brandstedt, 2012 [159]	The Malmo Diet and Cancer Study cohort, Sweden, men, born in 1923–1945	Case control	943 cases, 943 controls	Serum total calcium	Prostate cancer	OR: 1.34 (0.78–1.39) for highest versus lowest quartile	Matched on BMI, educational level, alcohol consumption, and smoking.
Schwartz, 2012 [160]	National Health and Nutrition Examination Survey III (NHANES III), USA, age 18+	Cohort	6707 at baseline, 49 events, 1069327 person-months	Serum total calcium	Prostate cancer mortality	HR: 1.50 (95% CI: 1.04–2.17) for every unit increase	Adjusted for age and BMI, serum albumin, and serum 25-OHD and account for survey weights and the complex sampling design of NHANES III
Salem, 2013 [161]	Iran, men, mean age 71.1 (cases) and 66.5 (controls)	Case control	194 cases, 317 controls	Serum total calcium	Prostate cancer	OR: 0.27 (0.12–0.59) for or highest versus lowest tertile	Adjusted for age, body mass index, occupation, educational level, smoking, alcohol, family history of prostate cancer, and sex hormones. Similar results with albumin-corrected calcium
